# Prevalence and Ergonomic Risk Factors for Musculoskeletal Pain Among Clinical Nurses in Türkiye: A Cross‐Sectional Study

**DOI:** 10.1155/prm/8412980

**Published:** 2026-08-05

**Authors:** Habip Balsak, Askeri Türken

**Affiliations:** ^1^ Department of Midwifery, Faculty of Health Sciences, Batman University, Batman, Türkiye, batman.edu.tr; ^2^ Department of Physical Medicine and Rehabilitation, Gazi Yaşargil Training and Research Hospital, University of Health Sciences, Diyarbakır, Türkiye, akdeniz.edu.tr

**Keywords:** clinical nurse, ergonomic, musculoskeletal pain, occupational health, workplace

## Abstract

**Background:**

Musculoskeletal pain (MSP) is among the most common occupational health problems for nurses. The purpose of this study was to evaluate the prevalence and severity of work‐related MSP in clinical nurses.

**Methods:**

This cross‐sectional and descriptive study was conducted with individuals who had been working as clinical nurses for at least 6 months. The study included 312 clinical nurses who were directly involved in patient care and met the eligibility criteria. Clinical nurses were assessed with the Ergonomic Risks Questionnaire (Ergo‐Nurse), and work‐related MSP was assessed with the Cornell Musculoskeletal Disorder Questionnaire (CMDQ). Spearman correlation, independent samples *t*‐test, and hierarchical regression analysis were used to determine the relevant factors.

**Results:**

Work‐related MSP is a major health problem in clinical nurses, with MSP severity ranging from low lumbar > back > neck. Hierarchical regression analysis showed that biomechanical factors were the most important ergonomic factor associated with work‐related MSP in clinical nurses (*R* ^2 Change^: 17.178 *p* < 0.05), and other ergonomic subdimensions did not contribute significantly to the model (*p* > 0.05). In the gender variable, being female was found to be a significant risk factor for work‐related MSP (*p* < 0.05).

**Conclusions:**

Biomechanical ergonomic risks and female sex were the main factors associated with work‐related MSP among clinical nurses. These findings highlight the need for targeted ergonomic interventions, safe patient‐handling practices, workload regulation, and institutional strategies to reduce musculoskeletal strain in clinical nursing settings.

## 1. Background

Healthcare employees are exposed to various risks and hazards in their work settings along with significant work‐related musculoskeletal pain (MSP) primarily as a result of ergonomic and psychosocial factors. It is reported that the prevalence of MSP among healthcare employees varies between 26% and 100% [1, 2]. The conditions in the work setting and many different variables might cause MSP to be detected at different rates. However, the common result of previous studies is that MSP might be an occupational healthcare concern that affects all healthcare employees if it is not managed well [1–3].

Nurses work in environments characterized by intense physical demands and multiple ergonomic risk factors, which position them as a high‐risk group for MSP [4, 5]. Their involvement in physically demanding tasks such as direct patient care, patient handling, and mobility assistance further increases exposure to biomechanical strain [6]. Clinical nurses (CNs), in particular, frequently experience MSP in the neck, back, and lower back due to repetitive activities, patient positioning, transportation, poor posture, and high physical workload [6, 7], all of which negatively affect their quality of life and job performance [8]. Recent systematic reviews reinforce this evidence, reporting high prevalence rates of work‐related musculoskeletal disorders among nursing populations. Clari et al. identified especially elevated rates in the lower back, neck, and shoulders [9]; Zare et al. highlighted the contribution of psychosocial stressors such as workload, insufficient support, and time pressure to upper‐limb disorders [10]; and Wang et al. emphasized that long weekly working hours, extended years of service, and female sex represent significant risk factors [11]. Collectively, these findings underscore the need to evaluate both physical and organizational determinants when examining MSP in CNs.

Ergonomic interventions come to the forefront as an effective method in reducing the prevalence of MSP among nurses. In a systematic review, nurses reported a reduction of 18%–72% in musculoskeletal injuries following ergonomic arrangements and education programs [12]. An experimental study that was conducted with operating room nurses reported that ergonomic interventions might reduce MSP in many parts of the body [13]. Studies conducted with intensive care nurses reported that approaches such as training programs, stretching exercises, and the use of assistive devices might be effective in reducing the risk and prevalence of MSP in body parts such as the ankle, hand/wrist, waist, neck, hip, and shoulder [14, 15]. The implementation of structured programs such as personalized ergonomic interventions was shown to be effective in improving posture and reducing pain intensity and pain‐related medication use [14, 15].

In conclusion, ergonomic factors have significant impacts on the prevalence of MSPs, and the selection of the right ergonomic interventions is of critical importance in the management of MSPs [8, 16, 17]. Although the literature emphasizes that ergonomic interventions are an effective method in the control of MSP in nurses, it also reports that the success of these interventions is limited [17]. For this reason, more studies are needed to ensure the integration of ergonomic approaches into professional practice and their long‐term success. Considering the diversity of ergonomic practices, it is necessary to determine the most effective interventions in the management of MSP and to develop new strategies specifically for CNs. In this context, a detailed definition of the individual and ergonomic factors that cause MSPs in CNs and the development of the right interventions are of great importance.

**FIGURE 1:**
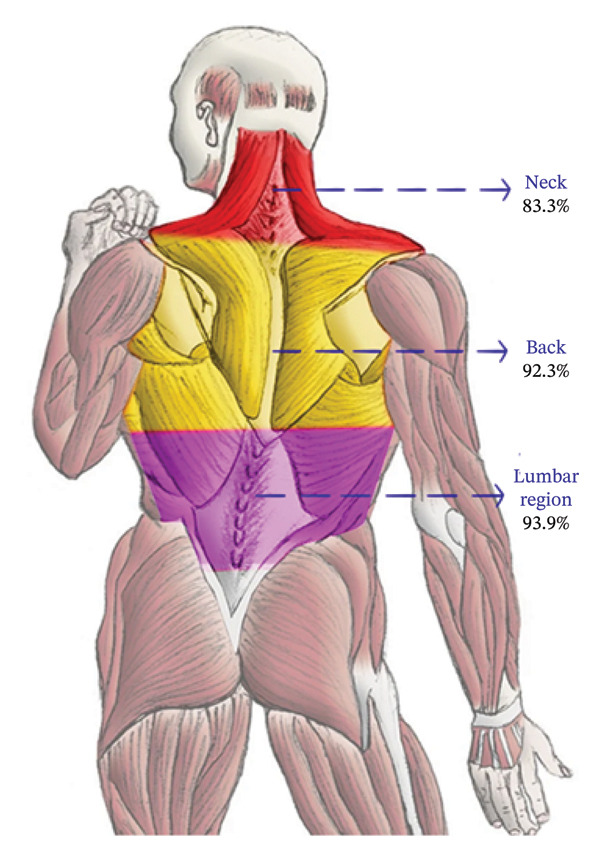
The most prevalent musculoskeletal pain regions among clinical nurses (*n*: 312).

The present study aimed to evaluate the prevalence and severity of CNs’ work‐related MSPs, determine the descriptive and ergonomic factors contributing to this condition, and uncover the order of importance of these factors. In this context, the study sought answers to the study questions justified by the literature stated below (Figure 1).

(Anatomical depiction of the most common back pain regions, illustrated by the authors.)

### 1.1. Study Question 1

 What is the prevalence and severity of work‐related MSP in CNs?
   In the literature, many studies conducted with healthcare staff and nurses report different levels of MSP. It is reported that these rates are in a wide range, ranging between 26% and 100% [1, 2]. Studies conducted on the level of these values, which vary depending on many different variables such as measurement tools and work setting, are limited in CN_S_. In this respect, defining the prevalence of work‐related MSP in CN nurses is important and must be addressed before determining the associated variables.

### 1.2. Study Question 2

 What is the relationship between work‐related MSP in CNs and demographics, work characteristics, and ergonomic factors?
   Work‐related MSP in healthcare employees and nurses has been associated with several variables in the literature, and the effects of ergonomic factors on this condition have been emphasized frequently [6, 8, 16]. To develop effective ergonomic interventions for CN_S_, it is necessary to identify the main determinants of work‐related MSP. Accurate identification of these factors plays critical roles in creating appropriate intervention strategies for CN_S_.

## 2. Methods

### 2.1. Study Design and Population

The study had a descriptive and cross‐sectional design. The study population consisted of CNs working in patient care units. Participants were eligible if they were actively employed as CNs, directly involved in routine patient care, had at least 6 months of clinical nursing experience, voluntarily agreed to participate, and completed the questionnaire form. Nurses working primarily in administrative, supervisory, or educational roles, such as responsible nurses, nurse managers, or training nurses, were not included in the study. In addition, nurses with less than 6 months of clinical nursing experience and incomplete questionnaire forms were excluded from the analysis.

The sample size of the study was determined by using the Epi Info Package Program. When the current literature was reviewed, it was detected that healthcare employees experienced MSP at rates between 26% [1] and 100% [2]. For this reason, to reach the highest sample size, the expected frequency was taken as 50%, and it was aimed to reach at least 164 people in the sample calculation to exceed the 80% confidence interval at a 5% error margin. As this study employed a convenience sampling method, CNs were invited to participate through face‐to‐face and online channels (social media, e‐mail, and WhatsApp groups). The survey form was applied to those who agreed to participate in the study by the researchers using online and face‐to‐face methods. As a result, the study was concluded with 312 nurses who met the study criteria. As a result of the study, a resample evaluation was made by considering the prevalence of independent variables (i.e., waist, neck, and back pain). In this respect, when the expected prevalence was taken as 83.3%, it was detected that the 97% confidence interval was exceeded with 312 people at a 5% error margin.

### 2.2. Data Collection

Data collection was performed with a data collection form that was prepared by the researchers. The data were collected by using the Data Collection Form which included sociodemographic data (age, sex, and marital status), and Introductory Data Form which included work characteristics (weekly working hours and number of shifts), the Cornell Musculoskeletal Discomfort Questionnaire (CMDQ) which evaluated work‐related MSP, and the Questionnaire Survey of Ergonomic Risks Among Nursing Workers (Ergo‐Nurse) which evaluated ergonomic working conditions of nurses.

### 2.3. CMDQ

Developed by the Cornell University Ergonomic Laboratory, CMDQ was used to evaluate the presence, frequency, severity, and work interference of MSP across 20 specific body regions, including the neck, right and left shoulders, back, right and left upper arms, waist, right and left forearms, right and left wrists, hips, right and left upper legs, right and left knees, right and left lower legs, and right and left feet [18, 19]. The Turkish version of the CMDQ, whose validity and reliability were confirmed by Erdinc et al., provides a comprehensive evaluation of musculoskeletal discomfort [20].

Pain frequency over the previous week was rated on a 5‐point Likert scale as “*Never*” (0), “*1-2 times last week*” (1.5), “*3-4 times last week*” (3.5), “*Once every day*” (5), and “*Several times every day*” (10). Pain severity was categorized on a 3‐point scale as “*Mild*” (1), “*Moderate*” (2), and “*Severe*” (3). The degree to which pain interferes with work was also rated on a 3‐point scale as “*Slightly*” (1), “*Moderately*” (2), and “*Highly*” (3).

For each body region, the Musculoskeletal Pain Score (MSPS) was calculated by multiplying frequency, severity, and work interference scores. Total scores ranged from 0, indicating no pain or discomfort, to 90, indicating severe pain and significant work interference. Higher scores reflect greater musculoskeletal discomfort and work interference. However, a score of zero in a body region is interpreted as indicating that there is no work‐related MSP in that region.

### 2.4. Questionnaire Survey of Ergonomic Risks Among Nursing Workers (Ergo‐Nurse)

Questionnaire Survey of Ergonomic Risks Among Nursing Employees (Ergo‐Nurse) was developed in English by Coluci and Alexandre to evaluate ergonomic risk factors in nurses [21]. The Turkish validity and reliability study of the scale was performed by Ercan et al. [22]. The scale, which consists of a total of 32 questions, has four sections (biomechanical factors (9 items), environmental factors/workplace (7 items), organizational factors (8 items), and psychosocial factors (8 items)). Ergo‐Nurse is answered in the form of a 10‐point Likert‐style and is scored separately for each section. An increase in the score indicates an increase in ergonomic risk.

### 2.5. Statistical Analysis

The data obtained in the study were analyzed by transferring them into the SPSS 24 package program. In the analysis of the data, descriptive statistical methods such as frequency, percentage, arithmetic mean, and standard deviation were used. The Spearman correlation analysis was used to evaluate the relationship between the continuous data. The normal distribution evaluation of neck, back, and waist pain that were determined as the independent variables of the study was evaluated with skewness and kurtosis scores. It was interpreted that these values between −2 and +2 provided normal distribution [23]. The independent samples *t*‐test was used to evaluate the work‐related neck, back, and back pain scores of categorical independent variables.

As a result of the correlation tests, the variables that were found to be significant (weekly working hours, sex, and Ergo‐Nurse scores) and the highest prevalence CMDQ scores of the neck, waist, and back regions were examined by using the hierarchical regression analysis. In creating the regression model, Ergo‐Nurse subdimension scores (ergonomic variables), sex, and weekly working hours (descriptive variables) were included, respectively. In this analysis, the most important variables affecting the CMDQ scores were determined. The multicollinearity in the regression models was evaluated with tolerance and variance inflation factor (VIF) values, and the autocorrelation in the error terms was analyzed with the Durbin–Watson statistics. As a result of the analysis, a *p* value below 0.05 was accepted as the significance criterion.

### 2.6. Ethics

Ethical approval was obtained from the Batman University Ethics Committee for the study (Date: 14.09.2022; Number: 2022/08‐20). The study was planned and conducted in line with the principles of the Declaration of Helsinki. Before the data were collected from the individuals who would participate in the study, the purpose, scope, method, and voluntary participation of the study were explained, informed consent was obtained from the participants, and their identity information was kept confidential.

## 3. Results

Table 1 shows the descriptive characteristics of the participants. Among the categorical variables, 59.0% of the participants were female and 41.0% were male. The majority of the participants were married (54.2%) and had a university‐level education (87.8%). In terms of income, 63.1% reported that their expenses exceeded their income. Most participants were employed in clinics or emergency units (67.9%), and the majority worked in shifts (84.3%).

**TABLE 1:** Descriptive characteristics.

Categorical variables	*n*	%
Sex		
Female	184	59.0
Male	128	41.0
Marital status		
Married	169	54.2
Single	143	45.8
Education		
University	274	87.8
Postgraduate	38	12.2
Income		
Greater than expenditure	20	6.4
Equal to expenditure	95	30.4
Less than expenditure	197	63.1
Work unit		
Clinics and Emergency	212	67.9
Intensive Care Unit	100	32.1
Shift Working		
Yes	263	84.3
No	49	15.7

**Continuous variables**	**Mean**	**SD**

Age	31.13	5.96
BMI (kg/m^2^)	23.99	3.60
Working period (years)	8.02	6.22
Weekly working hours	47.42	13.69
Monthly number of shifts	5.94	4.47

For the continuous variables, the participants’ mean age was 31.13 ± 5.96 years, their mean body mass index (BMI) was 23.99 ± 3.60 kg/m^2^, and their average working duration was 8.02 ± 6.22 years. Weekly working hours averaged 47.42 ± 13.69, while the monthly number of shifts was 5.94 ± 4.47.

Table 2 summarizes the prevalence, range, mean (±SD), and correlation values for MSP across various body regions, alongside the dimensions of ergonomic working conditions (Cornell and Ergo‐Nurse).

**TABLE 2:** The relationship between musculoskeletal pain and ergonomic working conditions and descriptive characteristics.

CMDQ and Ergo‐Nurse (*n*: 312)	Prevalence % (*n*)	Range	$\overset{¯}{x}\pm \text{SD}$	CMDQ and Ergo‐Nurse correlation^∗^			
				1	2	3	4
Neck	83.3% (260)	(0–90)	15.17 ± 17.99	0.456^∗∗^	0.351^∗∗^	0.388^∗∗^	0.384^∗∗^
Right shoulder	64.1% (200)	(0–90)	9.39 ± 15.10	0.459^∗∗^	0.333^∗∗^	0.320^∗∗^	0.366^∗∗^
Left shoulder	64.4% (201)	(0–90)	8.99 ± 14.95	0.398^∗∗^	0.272^∗∗^	0.277^∗∗^	0.326^∗∗^
Lumbar	93.9% (293)	(0–90)	24.11 ± 22.21	0.501^∗∗^	0.361^∗∗^	0.413^∗∗^	0.356^∗∗^
Back	92.3% (288)	(0–90)	22.83 ± 22.03	0.542^∗∗^	0.381^∗∗^	0.430^∗∗^	0.390^∗∗^
Right arm	60.5% (189)	(0–90)	7.74 ± 12.97	0.401^∗∗^	0.304^∗∗^	0.296^∗∗^	0.356^∗∗^
Left arm	58.6% (183)	(0–75)	6.81 ± 11.82	0.377^∗∗^	0.293^∗∗^	0.279^∗∗^	0.346^∗∗^
Right forearm	53.2% (166)	(0–75)	6.48 ± 11.68	0.365^∗∗^	0.279^∗∗^	0.270^∗∗^	0.325^∗∗^
Left forearm	52.5% (164)	(0–75)	5.74 ± 10.29	0.326^∗∗^	0.253^∗∗^	0.244^∗∗^	0.314^∗∗^
Right wrist	60.5% (189)	(0–90)	9.07 ± 15.42	0.384^∗∗^	0.270^∗∗^	0.272^∗∗^	0.303^∗∗^
Left wrist	57.6% (180)	(0–90)	7.43 ± 13.12	0.337^∗∗^	0.280^∗∗^	0.239^∗∗^	0.315^∗∗^
Hip	66.3% (207)	(0–90)	12.16 ± 19.28	0.323^∗∗^	0.309^∗∗^	0.281^∗∗^	0.310^∗∗^
Right calf	67.3% (210)	(0–90)	10.40 ± 15.78	0.367^∗∗^	0.327^∗∗^	0.276^∗∗^	0.349^∗∗^
Left calf	64.4% (201)	(0–90)	10.11 ± 15.48	0.353^∗∗^	0.316^∗∗^	0.262^∗∗^	0.350^∗∗^
Right knee	68.9% (215)	(0–90)	12.87 ± 18.10	0.377^∗∗^	0.299^∗∗^	0.276^∗∗^	0.322^∗∗^
Left knee	68.2% (213)	(0–90)	13.03 ± 18.60	0.371^∗∗^	0.282^∗∗^	0.269^∗∗^	0.307^∗∗^
Right leg	67.6% (211)	(0–90)	12.76 ± 18.37	0.340^∗∗^	0.255^∗∗^	0.315^∗∗^	0.332^∗∗^
Left leg	68.2% (213)	(0–90)	12.87 ± 18.39	0.361^∗∗^	0.283^∗∗^	0.335^∗∗^	0.361^∗∗^
Right foot	63.7% (199)	(0–90)	11.92 ± 18.02	0.351^∗∗^	0.287^∗∗^	0.322^∗∗^	0.338^∗∗^
Left foot	64.1% (200)	(0–90)	12.11 ± 17.53	0.360^∗∗^	0.290^∗∗^	0.306^∗∗^	0.350^∗∗^

**Ergo-Nurse**	**—**	**Min–Max**	$\overset{¯}{x}\pm \mathrm{SD}$	**1**	**2**	**3**	**4**

1. Biomechanics	—	(10–100)	71.11 ± 23.13	1.000	0.729^∗∗^	0.766^∗∗^	0.706^∗∗^
2. Workplace	—	(10–100)	68.71 ± 23.90		1.000	0.759^∗∗^	0.763^∗∗^
3. Organization	—	(10–100)	76.08 ± 22.23			1.000	0.760^∗∗^
4. Psychosocial	—	(10–100)	69.92 ± 22.68				1.000

^∗^Spearmen correlation.

^∗∗^ *p* < 0.05.

The three regions with the highest prevalence of MSP were the lumbar region (93.9%), back (92.3%), and neck (83.3%). The mean pain scores varied, with the highest in the lumbar region (24.11 ± 22.21), followed by the back (22.83 ± 22.03) and neck (15.17 ± 17.99). Correlation analyses revealed weak‐to‐moderate relationships between MSP scores and the dimensions of Ergo‐Nurse (biomechanics, workplace, organization, and psychosocial conditions), with correlation coefficients ranging from 0.244 to 0.542 (*p* < 0.05).

For the Ergo‐Nurse dimensions, the mean scores were as follows: biomechanics (71.11 ± 23.13), workplace (68.71 ± 23.90), organization (76.08 ± 22.23), and psychosocial factors (69.92 ± 22.68). Moderate correlations were observed among these dimensions, with coefficients ranging from 0.706 to 0.760 (*p* < 0.05).

As shown in Table 3, female participants reported significantly higher neck (*t* = 4.541, *p* < 0.001), back (*t* = 4.492, *p* < 0.001), and lumbar pain (*t* = 4.064, *p* < 0.001) compared to male participants. Additionally, those working 40 or more hours per week had significantly higher neck (*t* = −1.973, *p* = 0.049), back (*t* = −2.260, *p* = 0.025), and lumbar pain (*t* = −2.515, *p* = 0.012) compared to those working fewer hours. Lumbar pain was not significantly associated with working years (*t* = −1.959, *p* = 0.051).

**TABLE 3:** Descriptive characteristics and evaluation of neck, back, and lumbar pain.

Categorical variables		*n*	Neck		Back		Lumbar	
			Mean	S.D	Mean	S.D	Mean	S.D
Sex	Female	184	18.93	19.52	27.54	23.13	28.45	22.26
	Male	128	9.72	13.88	16.23	18.56	18.17	20.81
	Test values (*t* and *p*)		**4.541**	< **0.001**	**4.492**	< **0.001**	**4.064**	< **0.001**

Age	< 30	158	15.82	18.12	23.84	21.31	24.55	21.36
	≥ 30	154	14.55	17.91	21.82	22.76	23.68	23.09
	Test values (*t* and *p*)		0.617	0.538	0.787	0.432	0.338	0.736

Marital status	Married	169	16.35	18.83	23.09	22.59	24.68	22.84
	Single	143	13.80	16.94	22.54	21.44	23.43	21.50
	Test values (*t* and *p*)		1.237	0.217	0.211	0.833	0.486	0.627

Education	University	274	15.26	18.00	22.95	22.02	24.02	22.02
	Postgraduate	38	14.56	18.22	22.00	22.47	24.83	23.92
	Test values (*t* and *p*)				0.239	0.811	−0.207	0.836

Income	Income < expenditure	197	13.27	0.220	0.826	19.77	21.50	20.78
	Income ≥ expenditure	115	16.28	18.59	24.21	23.17	25.59	22.91
	Test values (*t* and *p*)		−1.412	0.159	−1.419	0.157	−1.538	0.125

Work unit	Clinics and Emergency	212	14.10	17.45	22.28	22.25	23.01	21.59
	Intensive Care Unit	100	17.47	18.98	23.98	21.66	26.32	23.38
			−1.533	0.126	−0.622	0.535	−1.217	0.225

Weekly working hours	< 40	162	13.27	16.72	20.15	21.00	21.08	20.67
	≥ 40	150	17.32	19.16	25.91	22.85	27.47	23.42
	Test values (*t* and *p*)		**−1.973**	**0.049**	**−2.260**	**0.025**	**−2.515**	**0.012**

Working year	≤ 9 years	206	15.36	18.31	22.34	21.33	22.42	21.05
	> 9 years	106	14.78	17.38	23.93	23.61	27.79	24.26
	Test values (*t* and *p*)		0.259	0.796	−0.575	0.566	−1.959	0.051

Shift working	Yes	263	14.93	17.36	23.34	22.34	24.22	22.29
	No	49	16.51	21.33	19.91	20.12	23.52	22.00
	Test values (*t* and *p*)		−0.552	0.581	0.943	0.346	0.196	0.845

*Note:* Back and lumbar pain. Bold values indicate statistically significant results at *p* < 0.05.

In contrast, variables such as age (neck: *t* = 0.617, *p* = 0.538; back: *t* = 0.787, *p* = 0.432; lumbar: *t* = 0.338, *p* = 0.736), marital status (neck: *t* = 1.237, *p* = 0.217; back: *t* = 0.211, *p* = 0.833; lumbar: *t* = 0.486, *p* = 0.627), an education level (neck: *t* = 0.220, *p* = 0.826; back: *t* = 0.239, *p* = 0.811; lumbar: *t* = −0.207, *p* = 0.836), income level (neck: *t* = −1.412, *p* = 0.159; back: *t* = −1.419, *p* = 0.157; lumbar: *t* = −1.538, *p* = 0.125), work unit (neck: *t* = −1.533, *p* = 0.126; back: *t* = −0.622, *p* = 0.535; lumbar: *t* = −1.217, *p* = 0.225), and shift work (neck: *t* = −0.552, *p* = 0.581; back: *t* = 0.943, *p* = 0.346; lumbar: *t* = 0.196, *p* = 0.845) did not demonstrate statistically significant differences in pain levels. These results suggest that gender and weekly working hours are critical factors in MSP, while other demographic and occupational variables showed no meaningful association.

In Model 1, the biomechanical subdimension significantly predicted neck pain (*β* = 0.368, *p* < 0.001). The psychosocial factor showed a weak, nonsignificant association (*β* = 0.161, *p* = 0.099). Psychosocial (*β* = 0.161, *p* = 0.099), organizational (*β* = −0.055, *p* = 0.598), and workplace (*β* = −0.023, *p* = 0.802) factors were not found to have a significant association with neck pain. Model 2 introduces gender as an additional factor. Being female was found to be a factor that increased work‐related neck pain (*β* = −0.158, *p* = 0.004). Weekly working hours did not significantly affect neck pain (*β* = 0.050, *p* = 0.342). The model’s fit improved slightly, with an *R* ^2^ change from 0.186 to 0.210, and this change was statistically significant (*F*‐change = 4.529, *p* < 0.05).

In Model 1, the biomechanical subdimension significantly predicted back pain (*β* = 0.460, *p* < 0.001). Psychosocial (*β* = 0.028, *p* = 0.769), organizational (*β* = 0.042, *p* = 0.679), and workplace (*β* = −0.026, *p* = 0.771) factors showed no significant relationships with back pain.

Model 2 shows that being female was a factor that increased work‐related back pain (*β* = −0.138, *p* = 0.009). Weekly working hours (*β* = 0.049, *p* = 0.349) did not significantly affect back pain. The model fit improved slightly, with an *R* ^2^ change from 0.245 to 0.264, and this change was statistically significant (*F*‐change = 3.363, *p* < 0.05).

In Model 1, it was found that the biomechanical subdimension was significantly related to low back pain (*β* = 0.459, *p* < 0.001). Psychosocial (*β* = 0.000, *p* = 1.000), organizational (*β* = 0.037, *p* = 0.729), and workplace (*β* = −0.005, *p* = 0.959) factors did not have significant effects on low back pain.

Model 2 showed that being female was a factor that increased work‐related low back pain (*β* = −0.114, *p* = 0.032). Weekly working hours (*β* = 0.066, *p* = 0.202) did not significantly affect low back pain. The model fit improved from *R* ^2^ = 0.236 to *R* ^2^ = 0.250, with a statistically significant change (*F*‐change = 2.894, *p* < 0.05).

Overall, the hierarchical regression analysis suggested that the biomechanical subdimension was consistently associated with work‐related neck, back, and lower back pain, while being female was a factor that increased work‐related MSP across all three types of pain.

## 4. Discussion

The study investigated the prevalence of work‐related MSP in CNs and evaluated the relationship between these disorders and ergonomic working conditions and demographic variables. A comprehensive MSP evaluation was performed in 20 different body regions using the CMDQ, and ergonomic conditions were assessed in four subdimensions using the Ergo‐Nurse scale. The study provides an original contribution to the literature by determining the ergonomic and descriptive factors associated with work‐related MSP in CNs and ranking these factors in order of importance. The absence of a study examining all body regions specifically in CNs further increases the originality and significance of the research.

The results uncovered a high prevalence of MSP, especially in the waist, back, and neck regions (93.9%, 92.3%, and 83.3%). Even in the forearm regions—where the lowest MSP prevalence was observed—pain rates reached 52.5%–53.5% (Table 2). These findings indicate that MSP is highly prevalent among. MSP is known to be common among healthcare employees, particularly in the waist and neck regions [24, 25]. However, prevalence rates vary across studies. For instance, in a Turkish hospital study, 79.3% of employees reported MSP, with the most affected areas being the back (61.5%), waist (55.6%), and neck (53.5%) [3]. Another study reported MSP in 26%–44% of clinical healthcare staff [1], while a separate investigation found that all nurses and dentists experienced pain in at least one region [2]. Such differences likely reflect variations in measurement tools, working conditions, and sample characteristics across studies. A key reason for the higher prevalence observed in the present study may be the specific composition of the sample. Unlike studies that include all nurses or broader healthcare professionals regardless of their actual clinical duties, the present study included only CNs actively engaged in direct patient care. Nurses working in administrative, supervisory, educational, or other nonclinical roles were excluded. Therefore, participants in this study were more likely to be exposed to physically demanding nursing activities, including patient lifting, repositioning, transferring, bedside care, prolonged standing, and awkward postures. This more clinically exposed sample may be associated with the higher MSP prevalence observed in the present study.

Four subdimensions of ergonomic variables showed weak and moderate positive correlations with MSP scores in all body regions. It is already known that ergonomic factors are associated with MSP in certain body regions in both the general working population [26] and among healthcare employees [27, 28]. For this reason, it is an expected result that poor ergonomic conditions are associated with MSP. However, different ergonomic conditions may be associated with MSP at varying levels [29, 30]. Therefore, in the present study, the variables associated with MSP were evaluated collectively through hierarchical regression analysis, and it was found that the biomechanical subdimension was the predictor variable with the highest beta coefficient among ergonomic factors, which may indicate that the effects of ergonomic risk factors on MSP are predominantly manifested through biomechanical exposures. It was also found that other ergonomic subdimensions did not make a significant contribution to the model (Table 4), possibly because psychosocial, organizational, and workplace‐related conditions exert their influence indirectly by shaping physical workload, posture, and movement patterns rather than acting as direct predictors; however, the present study did not test such indirect pathways through a formal mediation model. This finding is important as it suggests that biomechanical factors may play a primary role in association with MSP in CNs. In clinical nursing practice, these biomechanical exposures may be reflected in repeated patient lifting, repositioning, transferring, bending during bedside care, prolonged standing, patient mobilization, and maintaining awkward postures during medication administration, wound care, and other routine care procedures. Many previous studies showed that biomechanical factors such as prolonged postures, repetitive movements, and physically demanding tasks during work are associated with MSP among healthcare employees [8, 27, 31–33]. It was also reported that providing ergonomic support during physically straining activities such as lifting and bending might reduce MSP [34]. These results, consistent with the literature, offer important implications for understanding the factors associated with MSP in CNs. The significant and strong relationship observed between the biomechanical subdimension and neck, waist, and back pain emphasizes that physical workload and movement patterns must be optimized in clinical work settings, while other ergonomic dimensions may contribute cumulatively or indirectly through their impact on biomechanical load, although such mechanisms could not be examined within the scope of the current study.

**TABLE 4:** Evaluation of variables associated with work‐related neck, back, and low back pain by heirarchical regression.

		*β*	*t*	*p*	95% CI
Neck (Model 1)					
Ergonomic factors	Biomechanics	0.368	4.140	< 0.001	0.19/0.54
	Psychosocial	0.161	1.653	0.099	−0.03/0.35
	Organization	−0.055	−0.528	0.598	−0.25/0.14
	Workplace	−0.023	−0.250	0.802	−0.20/0.16
	Test values: *R* ^2^: 0.186; *R* ^2 Change *F*^: 17.178 *p* < 0.05				

Neck (Model 2)					
Ergonomic factors	Biomechanics	0.335	3.774	< 0.001	0.15/0.51
	Psychosocial	0.159	1.649	0.100	−0.03/0.34
	Organization	−0.069	−0.666	0.506	−0.27/0.13
	Workplace	−0.030	−0.331	0.741	−0.22/0.16
Other factors	Sex (1: Female; 2: Male)	−0.158	−2.939	0.004	−0.26/−0.05
	Weekly working (1: < 40; 2: > 40)	0.050	0.952	0.342	−0.06/0.16
	Test values: *R* ^2^: 0.210; *R* ^2 Change *F*^: 4.529 *p* < 0.05				

Back (Model 1)					
Ergonomic factors	Biomechanics	0.460	5.352	< 0.001	0.29/0.63
	Psychosocial	0.028	0.295	0.769	−0.18/0.23
	Organization	0.042	0.415	0.679	−0.17/0.25
	Workplace	−0.026	−0.292	0.771	−0.22/0.16
	Test values: *R* ^2^: 0.245; *R* ^2 Change *F*^: 23.659 *p* < 0.05				

Model 2 (Model 2)					
Ergonomic factors	Biomechanics	0.429	4.978	< 0.001	0.25/0.61
	Psychosocial	0.018	0.192	0.848	−0.18/0.22
	Organization	0.036	0.353	0.725	−0.16/0.25
	Workplace	−0.029	−0.332	0.740	−0.21/0.15
Other factors	Sex (1: female; 2: male)	−0.138	−2.612	0.009	−0.24/−0.03
	Weekly working (1: < 40; 2: > 40)	0.049	0.938	0.349	−0.06/0.17
	Test values: *R* ^2^: 0.264; *R* ^2 Change *F*^: 3.363 *p* < 0.05				

Lumbar (Model 1)					
Ergonomic factors	Biomechanics	0.459	5.313	< 0.001	0.28/0.63
	Psychosocial	0.000	0.000	1.000	−0.18/0.18
	Organization	0.037	0.347	0.729	−0.16/−0.25
	Workplace	−0.005	−0.052	0.959	−0.19/0.18
	Test values: *R* ^2^: 0.236; *R* ^2 Change *F*^: 22.808 *p* < 0.05				

Lumbar (Model 2)					
Ergonomic factors	Biomechanics	0.428	4.923	< 0.001	0.25/0.61
	Psychosocial	−0.004	−0.037	0.970	−0.19/0.18
	Organization	0.026	0.249	0.804	−0.16/0.25
	Workplace	−0.005	−0.061	0.951	−0.19/0.18
Other factors	Sex (1: female; 2: male)	−0.114	−2.159	0.032	−0.22/−0.01
	Weekly working (1: < 40; 2: > 40)	0.066	1.278	0.202	−0.03/0.17
	Test values: *R* ^2^: 0.250; *R* ^2 Change *F*^: 2.894 *p* < 0.05				

*Note:* Back and low back pain by hierarchical regression.

One of the most striking findings of the present study was that MSP levels in the neck, waist, and back regions were significantly higher among female participants than among males. Based on the results of the hierarchical regression analysis, gender emerged as the predictor variable with the highest beta coefficient after biomechanical factors. In a previous study examining upper extremity pain among healthcare employees, it was reported that upper extremity MSPs were significantly more common in women than in men and that this situation was associated with domestic workload [35]. Previous studies conducted in different sectors also confirm that work‐related MSP is generally higher in women [36–39]. Evidence suggests that this disparity is shaped by a combination of physical workload, ergonomic exposures, and psychosocial and cultural factors. Research has shown that perceived job exertion and age may moderate the relationship between gender and musculoskeletal disability and pain [36], while sex‐related differences in burnout have been linked to MSP [37]. Qualitative and quantitative studies further highlight the role of cultural expectations, coping strategies, and gendered work roles in shaping women’s experiences of musculoskeletal symptoms at work [38]. Investigations among female industrial workers also demonstrate high MSP prevalence in relation to awkward postures, repetitive tasks, and adverse working environments [39, 40].

These findings underscore MSP as a major concern in occupational settings with a high proportion of female workers, including the health sector. Accordingly, alongside general ergonomic improvements, gender‐sensitive workplace interventions and institutional policies addressing physical workload, psychosocial stressors, and task design should be developed and implemented to mitigate MSP among female employees.

It was found that working more than 40 h per week was associated with higher levels of neck, waist, and back MSP in CNs. A meta‐analysis similarly reported that long working hours increase MSP risk [41]. Although the standard weekly working time in Turkey is 40 h, CNs may occasionally work beyond this limit for additional payment, and the extent of overtime in the study setting is not fully known. The lack of a significant contribution of weekly working hours in the regression model may therefore reflect the limited presence of overtime within the sample. However, considering the demanding working conditions of CNs, extended work hours, reduced rest periods, and cumulative physical workload may be associated with increased musculoskeletal strain. For this reason, regulating working hours and ensuring adequate rest intervals may be an important strategy for reducing MSP.

In the present study, it was also found that variables such as age, marital status, education level, income level, work unit, and shift system did not have a significant relationship with MSP. This situation might be evaluated as a natural result of the limited influence of demographic and organizational factors in a homogeneous occupational group such as CNs. Also, the effect of individual differences and the multifactorial structure of MSP support these findings. The results of the study uncovered that focusing on biomechanical factors, working conditions, and female employees rather than such variables may be more strongly associated with preventing MSP.

## 5. Conclusion and Recommendations

The results of this study show that MSP is highly prevalent and represents a significant occupational health concern among CNs. MSP was frequently reported in more than one body region, with the neck, back, and waist identified as the most common and severe areas. These findings highlight the need for targeted strategies to reduce MSP in clinical settings.

Ergonomic factors—especially the biomechanical subdimension—were strongly associated with MSP. The effects of other ergonomic and demographic variables were more limited. MSP was also more common in female CNs, which may reflect gender‐related workload and physical demands. Extended working hours and insufficient rest periods may be associated with increased musculoskeletal strain.

Practical implications are clear. Ergonomic adjustments, regulated working hours, and supportive policies for female CNs are essential to reduce MSP. In line with the main findings, practical interventions should focus primarily on reducing biomechanical load during routine nursing tasks. Administrators should prioritize safe patient‐handling protocols, use of assistive transfer devices, adjustment of bed and equipment height during bedside care, and short ergonomic training programs focused on lifting, bending, transferring, repositioning, and prolonged standing. Workload planning, adequate staffing, microbreaks during long shifts, and task rotation in high‐care‐burden units should also be considered to reduce cumulative musculoskeletal strain. Further studies in larger samples may support long‐term and sustainable prevention strategies.

## 6. Limitations

Although this study provides valuable insights into the prevalence of work‐related MSP and its associated factors among CNs, several considerations should be noted when interpreting the results. Firstly, the cross‐sectional design does not allow for conclusions about causality. Data were collected through self‐report instruments, which may introduce recall bias or social desirability tendencies, and this may have influenced how participants reported their symptoms and work conditions.

Ergonomic risk factors were assessed through self‐reported perceptions rather than direct observational tools, which may limit the level of objectivity but still provides useful information about nurses’ perceived ergonomic risks. In addition, the study employed a convenience sampling method, which may reduce the representativeness of the sample and should be considered when generalizing the findings.

The study was also conducted in a single region, which may limit the applicability of the results to nurses working in different institutional or cultural contexts. Although MSP appeared more common among female nurses and may relate to gender roles or domestic workload, these aspects were not explored in depth and could be addressed in future studies. Future studies using broader samples and complementary ergonomic assessment methods may help strengthen the generalizability and interpretation of these findings.

**Nomenclature**
MSP: Musculoskeletal pain
CN_s_
: Clinical nurses

## Author Contributions

Study conception and design: all authors. Data collection: all authors. Data analysis and interpretation: all authors. Drafting of the article: all authors. Critical revision of the article: all authors contributed equally.

## Funding

The authors received no financial support for the research, authorship, and/or publication of this article.

## Ethics Statement

All procedures performed in studies involving human participants were in accordance with the ethical standards of the institutional and/or national research committee and with the 1964 Helsinki Declaration and its later amendments or comparable ethical standards. Ethical approval was obtained from the Batman University Ethics Committee (Date: 14.09.2022; Decision no: 2022/08‐20). Consent was obtained from all participants included in the study.

## Consent

Please see the Ethics Statement.

## Conflicts of Interest

The authors declare no conflicts of interest.

## Data Availability

The data that support the findings of this study are available from the corresponding author upon reasonable request.
